# Improved ensemble of differential evolution variants

**DOI:** 10.1371/journal.pone.0256206

**Published:** 2021-08-20

**Authors:** Juan Yao, Zhe Chen, Zhenling Liu

**Affiliations:** 1 College of Informatics, Huazhong Agricultural University, Wuhan, Hubei, China; 2 School of Computer Science, China University of Geosciences, Wuhan, Hubei, China; 3 Hubei Key Laboratory of Intelligent Geo-Information Processing, China University of Geosciences, Wuhan, Hubei, China; 4 Network Management Department, Wuhan Information Center of Real Estate, Wuhan, Hubei, China; Universidad de Guadalajara, MEXICO

## Abstract

In the field of Differential Evolution (DE), a number of measures have been used to enhance algorithm. However, most of the measures need revision for fitting ensemble of different combinations of DE operators—ensemble DE algorithm. Meanwhile, although ensemble DE algorithm may show better performance than each of its constituent algorithms, there still exists the possibility of further improvement on performance with the help of revised measures. In this paper, we manage to implement measures into Ensemble of Differential Evolution Variants (EDEV). Firstly, we extend the collecting range of optional external archive of JADE—one of the constituent algorithm in EDEV. Then, we revise and implement the Event-Triggered Impulsive (ETI) control. Finally, Linear Population Size Reduction (LPSR) is used by us. Then, we obtain Improved Ensemble of Differential Evolution Variants (IEDEV). In our experiments, good performers in the CEC competitions on real parameter single objective optimization among population-based metaheuristics, state-of-the-art DE algorithms, or up-to-date DE algorithms are involved. Experiments show that our IEDEV is very competitive.

## Introduction

Differential evolution (DE), a type of population-based metaheuristic, is reliable and powerful for global numerical optimization. DE incorporates mutation, crossover and selection operators to move population gradually toward a global optimum [1]. At beginning of execution, target vectors ${\vec{x}}_{i,0}=\left(x_{1,i,0},x_{2,i,0},\ldots ,x_{D,i,0}\right)$ (*i* = 1, 2, …, *NP*), where *NP* denotes population size and *D* is dimensionality, are initialized randomly. Then, mutant vectors ${\vec{v}}_{i,g}$ are produced based on target vectors ${\vec{x}}_{i,g}$ by mutation in a generation *g*. Different mutation strategies are used in different DE algorithms. Three widely used mutation strategies—DE/rand/1, DE/best/1, and DE/current-to-best/1—are listed below for example,

$$\begin{array}{c} {\vec{v}}_{i,g}={\vec{x}}_{r1,g}+F\cdot \left({\vec{x}}_{r2,g}-{\vec{x}}_{r3,g}\right), \end{array}$$ (1)

$$\begin{array}{c} {\vec{v}}_{i,g}={\vec{x}}_{best,g}+F\cdot \left({\vec{x}}_{r1,g}-{\vec{x}}_{r2,g}\right), \end{array}$$ (2)

and

$$\begin{array}{c} {\vec{v}}_{i,g}={\vec{x}}_{i,g}+F\cdot \left({\vec{x}}_{best,g}-{\vec{x}}_{i,g}\right)+F\cdot \left({\vec{x}}_{r1,g}-{\vec{x}}_{r2,g}\right). \end{array}$$ (3)

In the equations, the distinct integers different from *i*—*r*1, *r*2, and *r*3—are randomly chosen from the range [1, *NP*]. *F* is the scaling factor, while ${\vec{x}}_{best,g}$ denotes the individual with the best fitness in the generation *g*. After mutation, crossover is executed based on ${\vec{x}}_{i,g}$ and ${\vec{v}}_{i,g}$ to generate trial vectors ${\vec{u}}_{i,g}=\left(u_{1,i,g},u_{2,i,g},\ldots ,u_{D,i,g}\right)$. Binomial crossover,

$$\begin{array}{c} u_{j,i,g}=\{\begin{array}{cc} v_{j,i,g}, & if\;rand(0,1)\leq Cr\;or\;j=randn(i),\\ x_{j,i,g}, & otherwise, \end{array} \end{array}$$ (4)

is widely used in DE algorithms. In Eq 4, *Cr* ∈ [0, 1] is the crossover rate, and *randn*(*i*) is an integer randomly generated from the range [1, *NP*] to ensure that ${\vec{u}}_{i,g}$ has at least one component from ${\vec{v}}_{i,g}$. In selection,

$$\begin{array}{c} {\vec{x}}_{i,g+1}=\{\begin{array}{cc} {\vec{u}}_{i,g}, & if\;f\left({\vec{u}}_{i,g}\right)\leq f\left({\vec{x}}_{i,g}\right),\\ {\vec{x}}_{i,g}, & otherwise, \end{array} \end{array}$$ (5)

where $f({\vec{u}}_{i,g})$ and $f({\vec{x}}_{i,g})$ represent fitness of ${\vec{u}}_{i,g}$ and ${\vec{x}}_{i,g}$, respectively. By this means, ${\vec{u}}_{i,g}$ competes with ${\vec{x}}_{i,g}$ for survival. The winner becomes the target vector of the next generation, ${\vec{x}}_{i,g+1}$.

For years, DE is improved in various ways. Recently, methods for improving DE include

- The improved trial vector generation strategies [2–10]
- The hybridizations of DE and other techniques [7, 9, 11–17]; and
- The developed types of ensemble of different combinations of operators [18–27]

It can be seen that ensemble is one of the active type of method for improving DE. All the types of ensemble listed above are be further introduced in our section for related work. Essentially, ensemble of different combinations of DE operators still belongs to DE algorithm and so called ensemble DE algorithm in this paper.

The motivation of this paper is given below. A number of measures have been used in DE algorithms. However, most of the measures need revision for fitting ensemble DE algorithm. Meanwhile, although ensemble DE algorithm may show better performance than each of its constituent algorithms, there still exists the possibility of further improvement on performance with the help of revised measures.

In this paper, we enhance Ensemble of Differential Evolution Variants (EDEV) [21], an ensemble DE algorithm, by measures listed below. Firstly, we revise the organization of optional external archive in one of the constituent DE algorithms of EDEV—JADE [28]. In detail, when JADE is used as a constituent algorithm, the individuals eliminated from other constituent algorithms are also sent to optional external archive. Then, we add Linear Population Size Reduction (LPSR), the widely used technique in the field of DE, into algorithm. Under the control of LPSR, population size is linearly reduced. Finally, we implement the Event-Triggered Impulsive (ETI) control, which is firstly introduced from control theory to DE by [12]. Here, the ETI control is implemented to the two inefficient constituent DE algorithms in ensemble confirmed by comparison at intervals. That is, after each time of performance comparison, not only the best performer among the three constituent algorithms as before, but also the other two constituent algorithms, are pointedly treated. Thus, we obtain our Improved Ensemble of Differential Evolution Variants (IEDEV). Our experiments are based on the CEC 2013, 2014 and 2017 benchmark testing suites. Nine peers are involved in experiment. Experimental results show that our IEDEV is competitive.

The rest of the paper is organized as follows. In Section II, related work is presented. In Section III, our IEDEV is given. Then, experimental results are shown and analyzed in Section IV. Finally, a conclusion and a prospect are dealt with in Section V.

## Related work

Here, related work includes two aspects. Firstly, we list existing ensemble DE algorithms including EDEV. Then, we review existing techniques for improving DE, including the measures taken by us—optional external archive, LPSR, and the ETI control.

In SaDE [29], each individual make a choice from multiple mutation strategies based on previous experiences of generating promising solutions. In EPSDE [30], recorded successful combinations of strategy and parameter are employed at high probability. In mDE-bES [31], population is divided into subpopulations. Each subpopulation are different in operators. After every certain number of generations, individuals are exchanged between subpopulations. MPEDE [32] has three mutation strategies. There are three equally sized smaller indicator subpopulations and one much larger reward subpopulation in population of MPEDE. Each constituent mutation strategy controls one indicator subpopulation. After every certain number of generations, the current best performing mutation strategy will be determined according to the ratios between fitness improvements and consumed function evaluations. Then, the reward subpopulation will be allocated dynamically to the determined best performing mutation strategy. In sTDE-dR [18], population is clustered in multiple tribes in which different mutation and crossover strategies are utilized. In each tribe, scaling factor and crossover rate are controlled by a different adaptive scheme. The mean success of each tribe is used to calculate the participation ratio for the next generation. In L-SHADE-SPA [33], there are two combinations of setting for both the parameter for mutation *F* and the one for crossover *CR*. L-SHADE-SPACMA [33] is a hybridization framework between LSHADE-SPA and a modified version of CMA-ES. In [34], a memetic framework for solving large-scale global optimization problems is proposed. In the framework, L-SHADE-SPA is used for global exploration, while a modified version of multiple trajectory search is used for local exploitation. In EL-SHADE-SPACMA [35], based on L-SHADE-SPACMA, the greediness of the mutation strategy is changed to be dynamic. Furthermore, another directed mutation strategy is integrated into framework. In the DE algorithm proposed by [19], both fitness landscapes and performance history of the operators to dynamically selecting the most suitable operator. In AMECoDEs [20], two elites-guided trial vector generation strategies may be both employed for each target vector in a generation to generate two trial vectors accordingly. In fact, only the better one can participate in selection. EDEV [21] consists of three highly popular and efficient DE algorithms—JADE [28], CoDE [1], and EPSDE. Similar with population of MPEDE, there are three indicator subpopulations and a reward subpopulation in population of EDEV. Also, the competition mechanism for the four subpopulations are similar with that in MPEDE. MLCC framework [22] implements a parallel structure with the entire population simultaneously monitored by multiple DE algorithms assigned to different layers. A target vector can store, utilize and update its evolution information in different layers. EMMSIQDE [23] is quantum-inspired DE based on mixing multiple strategies. In detail, a new multipopulation mutation evolution mechanism is designed. Meanwhile, the feasible solution space transformation strategy is used. IPOQEA [24] is ensemble based on quantum evolutionary algorithm and particle swarm optimization. This algorithm is proposed for gate allocation of airport. MSQCCEA [25] is an improved quantum-inspired cooperative co-evolution algorithm based on combining the strategies of cooperative co-evolution, random rotation direction and Hamming adaptive rotation angle. Also, the algorithm is proposed for gate allocation. HMCFQDE [26] is ensemble of quantum evolutionary algorithm(QEA) and cooperative coevolution evolutionary algorithm, which is based on a new hybrid mutation strategy consisting of local neighborhood mutation and SaNSDE. The coastal ship path planning model based on the optimized deep Q network (DQN) algorithm [27] is proposed for path planning in the field of coastal ships. In essence, the model is ensemble of environment status information and the DQN algorithm. The former provides training space for the latter.

JADE is a famous DE algorithm based on optional external archive. SHADE [36], which is also base on optional external archive, is revised from JADE and has many variants. For example, L-SHADE-SPA, L-SHADE-SPACMA, EL-SHADE-SPACMA, L-SHADE-RSP and EB-L-SHADE are all variants of SHADE. Under the control of the ETI scheme [12], stabilizing impulses and destabilizing impulses are executed based on the event-triggered mechanism. In fact, destabilizing impulses are essentially partial restarts. In [12], the ETI scheme is used in ten DE algorithms. The adaptive social learning strategy [14] can extract the neighborhood relationship information of individuals. Multi-topology-based DE [15], which is based on multiple population topologies, the individual-dependent adaptive topology selection scheme, and the topology-dependent mutation strategy, can utilize the information derived from the differences in fitness. Since [37], LPSR, which means linear decrease in population size, began to be employed in DE algorithms. Recently, LPSR is at least employed in [7, 9, 34, 35, 38].

## Our proposed algorithm

Firstly, we discuss EDEV, optional external archive, the ETI control, and LPSR for improvement. Then, we propose our IEDEV based on discussion in this section.

### Discussion on the involved methods

#### EDEV

EDEV [21] is a powerful ensemble DE algorithm. There are three constituent DE algorithms—JADE [28], CoDE [1], and EPSDE [30]—in EDEV. Population of EDEV is partitioned into four subpopulations, including three indicator subpopulations and a reward subpopulation. Each of the three constituent DE algorithms in EDEV owns an indicator subpopulation. After every predefined generations, the most efficient constituent DE algorithm is determined based on comparison. Then, the reward subpopulation is assigned to the determined one as an extra reward. In detail, at the beginning of execution, the reward subpopulation is allocated to one of the three constituent algorithm randomly. At intervals, the most efficient DE algorithm *A*_*k*_ (*k* = 1, 2, 3) is determined by

$$\begin{array}{c} A_{k}=argmax_{k=1,2,3}\frac{\Delta f_{k}}{\Delta FES_{k}}, \end{array}$$ (6)

where Δ*f*_*k*_ denotes improvement of the *k*th constituent algorithm in fitness and Δ*FES*_*k*_ indicates consumed function evaluations of the *k*th constituent algorithm. After that, the reward subpopulation is reallocated to the most efficient DE algorithm. More details of EDEV can be seen in [21].

It can be seen that there exists a paradox in EDEV. In detail, the best performing constituent algorithm obtains the reward, while the rewarded constituent algorithm may continue performing best with the help of the reward. In fact, no measure is adopted for the other two constituent algorithms to enhance them.

### Optional external archive

In JADE, SHADE and the most variants of SHADE, individuals eliminated by selection are all sent to the archive. However, the archive never accepts duplicated individuals. Furthermore, when the archive is full, randomly chosen individuals in it are replaced by new comers. The above method to organize the archive is simple and effective for maintaining diversity to resist stagnation.

In EDEV, the constituent algorithm—JADE—employs the archive. According to [28], the paper proposed JADE, the motivation of applying the archive is to give more choices when selecting individual for difference in mutation. Thus, diversity can be better maintained. In EDEV, compared with just using individuals eliminated from JADE, using individuals eliminated from all the three constituent algorithms to organizing the archive may be a better method for maintaining diversity.

#### The ETI control

The ETI control, which comes from the field of control theory, can be widely used in DE algorithms. The update rate (*UR*) of the population in the current generation needs to be observed during execution. *UR* is illustrated by Eq 7

$$\begin{array}{c} UR=\frac{UP}{NP}, \end{array}$$ (7)

where *NP* is the population size, and *UP* is the number of individuals updated in the current generation. When *UR* begins to decrease, for enhancing exploitation, stabilizing impulses drive the individuals with lower rankings in the current population to approach the individuals with better fitness values. When *UR* drops to zero or stabilizing impulses fail to take effect, to improve exploration, destabilizing impulses randomly adjust the positions of the individuals with lower rankings in the current population within the area of the current population. More details of stabilizing impulses and destabilizing impulses can be found in [12]. The ranking of each individual for exposed to the two types of impulses is given below. For ${\vec{x}}_{i,g}$,

$$\begin{array}{c} R_{i,g}={\tilde{R}}_{i,g}+{\bar{R}}_{i,g}, \end{array}$$ (8)

where ${\tilde{R}}_{i,g}$ denotes the ranking of ${\vec{x}}_{i,g}$ according to fitness and ${\bar{R}}_{i,g}$ denotes the ranking of that based on the number of consecutive stagnation generation. In the experiments of [12], the ETI control is applied on many DE algorithms including JADE, CoDE, and EPSDE and leads to significant improvement. In fact, the three DE algorithms are constituent algorithms of EDEV.

Although the ETI control can be widely used in DE algorithms, so far, it can be hardly seen that the technique is implemented in ensemble DE algorithm. In ensemble DE algorithm, the cooperation of different combinations of operators makes that the simple scheme for calling the two types of impulses in [12] becomes invalid. At least, Eq 7 cannot be valid for EDEV to control the two types of impulses because, in a moment, the three constituent DE algorithms may be different in state. For example, when one constituent algorithm leads to little update and need impulses, the other two constituent algorithms may be still active. In this case, *UR* may still not decrease, or even increase. Therefore, the two types of impulses cannot executed at all. In brief, a new scheme for calling the two types of impulses need be proposed for implementing the ETI control in EDEV.

#### LPSR

So far, LPSR has many applications in the field of DE. For example, L-SHADE [37], L-SHADE-EpSin [39], jSO [2], L-SHADE-SPACMA [33], L-SHADE-RSP [7], EAGDE [38], and EB-L-SHADE [40] are famous DE algorithms with LPSR. The algorithms are all good performers in competitions of real parameter single objective optimization among population-based metaheuristics held by the series of IEEE Congress on Evolutionary Computation (CEC). It can be seen that LPSR is useful for improving solution.

### IEDEV proposed by us

As mentioned before, in EDEV, no measure is adopted for the two constituent algorithms worse in performance to enhance them. Provided that another strategy, e.g., the EIT control, is employed the two constituent algorithms, competition among all the three constituent algorithms may become more intense than before. In this case, reallocation of the reward subpopulation becomes more frequent. Thus, algorithm performance may be further improved.

In our IEDEV, firstly, organization of optional external archive is changed. As mentioned before, individuals eliminated from all the three constituent DE algorithms are all sent to the archive. Moreover, the ETI control is applied in the two constituent DE algorithms not best in efficiency. In detail, after each time of reallocation of the reward subpopulation to the best performer among the three constituent DE algorithms, the ETI control is implemented in the other two ones. Here, the parameters for the ETI control, such as *UR* and *R*_*i*,*g*_, are computed just based on individuals in constituent DE algorithm. By this means, all constituent DE algorithms are all treated by different means according to their rank in comparison. That is, the most efficient constituent algorithm still obtains the reward subpopulation, while the other two constituent algorithms are supported by the ETI control. In addition, LPSR is applied here. That is, during the course of run, population size is decreased linearly.

The pesudo-code of our IEDEV is given in Algorithm 1. Nevertheless, the change in organization of optional external archive for JADE is not reflected by Algorithm 1 directly. In our algorithm, the simple multiple relation between generations and function evaluations in EDEV is not used any more. As shown in Step 7, the main loop is counted by function evaluations but not generations as EDEV any more.

**Algorithm 1** The pseudo-code of L-ETI-EDEV

**Input**:

*NP*_*max*_, the maximum value of *NP*

*NP*_*min*_, the minimum value of *NP*

*MaxFES*, the maximum number of function evaluations;

λ_*k*_ (*k* = 1, 2, 3, 4), the proportion between size of the *k*th subpopulation and *NP*

*ng*, interval for determining the most efficient constituent DE algorithm

**Parameter**:

*NP*, population size;

*A*_*k*_ (*k* = 1, 2, 3), the *k*th constituent DE algorithm (*A*_1_, *A*_2_, and *A*_3_ are JADE, CoDE, and EPSDE, respectively)

*FES*, fitness evaluations

*FES*_*k*_ (*k* = 1, 2, 3), consumed fitness evaluations of *A*_*k*_

1: Initialize population, *pop*

2: Set Δ*f*_*k*_ = 0 and Δ*FES*_*k*_ = 0 (*k* = 1, 2, 3)

3: Randomly divide *pop* into four subpopulations, *pop*_*k*_ (*k* = 1, 2, 3, 4), whose size is *NP*_*k*_ = λ_*k*_ ⋅ *NP*

4: Randomly select a integer *m* from [1, 3].

5: Let *pop*_*m*_ = *pop*_*m*_ ∪ *pop*_4_ and *NP*_*m*_ = *NP*_*m*_ + *NP*_4_

6: *FES* = *NP*, *r* = 0, and *r*′ = 0

7: **while** *FES* <= *MaxFES* **do**

8:  **for** *k* = 1 to 3 **do**

9:   Execute *A*_*k*_ on *pop*_*k*_

10:   calculate Δ*f*_*k*_ and Δ*FES*_*k*_

11:  **end for**

12:  $FES=FES+\sum_{k=1}^{3}\Delta FES_{k}$

13:  *pop* = ∪_*k* = 1,2,3_ *pop*_*k*_

14:  *r*′ = *r* and *r* = *mod*(*MaxFES*, *ng* ⋅ *NP*)

15:  **if** *r*′ > *r* **then**

16:   Determine the most efficient constituent DE algorithm *A*_*m*_ by Eq 6

17:  **end if**

18:  Randomly divide *pop* into four subpopulations, *pop*_*k*_ (*k* = 1, 2, 3, 4), whose size are *NP*_*k*_ = λ_*k*_ ⋅ *NP*

19:  Let *pop*_*m*_ = *pop*_*m*_ ∪ *pop*_4_ and *NP*_*m*_ = *NP*_*m*_ + *NP*_4_

20:  Add the ETI control into the two constituent DE algorithms other than *A*_*m*_

21:  *NP* = ((*NP*_*min*_ − *NP*_*max*_)/*MaxFES*) ⋅ *FES*) + *NP*_*max*_

22: **end while**

23: Report solution

## Experimental study

In the section, we list and compare computational results of the proposed algorithm and other algorithms. Firstly, we describe setup in experiment. Then, an experiment based on the CEC 2013 benchmark testing suite is executed to investigate the effect of each of the measures taken by us. After that, a comparative comparison against existing algorithms is executed based on the CEC 2014 and 2017 benchmark testing suites. In detail, we compare out IEDEV with EBOwithCMAR [41], jSO, L-SHADE-SPACMA, ETI-JADE, L-SHADE-RSP, EDEV, EAGDE, EB-L-SHADE, and NDE [9]. Among the peers, at least L-SHADE-SPACMA, ETI-JADE, L-SHADE-RSP, EB-L-SAHDE employ optional external archive, while ETI-JADE implement the ETI control. The all peers except EBOwithCMAR, ETI-JADE, and EDEV are all based on LPSR.

### Experimental settings

Settings of all the algorithms are shown in Table 1. Here, *D* denotes dimensionality, while *MaxFES* represents maximum number of function evaluations. The parameters for IEDEV are further explained as below. *NP*_*max*_ and *NP*_*min*_ is for LPSR. λ_1_, λ_2_, and λ_3_ are inherited from EDEV to define ratio of each constituent DE algorithm, while *LN* and *UN* are redefined by us for the ETI control. Details can be found in the last subsection of the previous section. In all experiments, *MaxFES* is set 10000 ⋅ *D*. We use two non-parametric statistical hypothesis tests to compare and analyze solution. In detail, the Friedman test is used for multiple comparison, while the Wilcoxon’s rank sum test for pairwise comparison.

**Table 1:** Settings for algorithms.

Algorithm	Parameters
EBOwithCMAR	*NP*_1,*max*_ = 18 ⋅ *D*, *NP*_1,*min*_ = 4, *NP*_2,*max*_ = 46.8 ⋅ *D*, *NP*_2,*min*_ = 10, *NP*_3_ = 4 + 3 ⋅ *log*(*D*), *H* = 6, *σ* = 0.3, *CS* = 200 when *D* = 30, *CS* = 300 when *D* = 100, *prob*_*ls*_ = 0.1 and *cfe*_*ls*_ = 0.25 ⋅ *MaxFES*
jSO	$NP_{max}=25\cdot log\left(D\right)\cdot \sqrt{D}$, *NP*_*min*_ = 4 *p*_*max*_ = 0.25, $p_{min}=\frac{p_{max}}{2}$, |*A*| = 1404, H = 5
L-SHADE-SPACMA	*NP*_*max*_ = 18 ⋅ *D*, *Pbest* = 0.11, *H* = 1.4, *Arc*_*rate* = 5, *F*_*CP*_ = 0.5, *c* = 0.8, *T* = 0.5 ⋅ *MaxFES*
ETI-JADE	*NP* = 100, *LN* = 1, *UN* = *NP*, *μ*_*F*_ = 0.5, *μ*_*CR*_ = 0.5, *c* = 0.1, |*A*| = 100, and *p* = 0.05 [12]
L-SHADE-RSP	$NP_{max}=D^{\frac{2}{3}}\cdot 75$, *NP*_*min*_ = 4, |*A*| = *NP*, *H* = 5, and *k* = 3 [7]
EDEV	*NP* = 60 when *D* = 30, *NP* = 100 when *D* = 50, 100, λ_1_ = λ_2_ = λ_3_ = 0.1, and *ng* = 20 [21]
EAGDE	*NP*_*max*_ = 180 when *D* = 30, *NP*_*max*_ = 400 when *D* = 100, *NP*_*min*_ = 12, p = 0.1
EBL-SHADE	*NP*_*max*_ = 18 ⋅ *D*, *NP*_*min*_ = 4, *Pbest* = 0.11, *EDE*_*best* = 0.10, |*A*| = 1.4
NDE	*NP*_*max*_ = 10 ⋅ *D*, *NP*_*min*_ = 5, *μ*_*F*_ = 0.5, *μ*_*CR*_ = 0.5, *gm* = 10, and *c* = 0.1 [9]
IEDEV	*NP*_*max*_ = 300, *NP*_*min*_ = 4 λ_1_ = λ_2_ = λ_3_ = 0.1, *ng* = 20, *LN* = 1, and *UN* = *NP*

### Experiment to investigate the effect of our measures

This experiment is based on the CEC 2013 benchmark testing suite which consists of 28 benchmark functions. In this experiment, *D* is set 30. For each function in the suite, EDEV, IEDEV-1 (EDEV changed in organization of the archive), IEDEV-2 (EDEV with the ETI control), IEDEV-3 (EDEV with LPSR), and our IEDEV are all executed 30 times, respectively. Results are given in Table 2. Then, in Table 3, we lists Friedman test ranks based on the results in Table 2. From the table, it can be seen that p-value is less than 0.05. That is, there exists significant difference on performance. According to Table 3, each measure taken by us enhances EDEV. Therefore, our IEDEV which integrates all the three measures performs much better than EDEV.

**Table 2:** Result of the experiment to investigate the effect of measures.

Function	Average (standard deviation) of algorithms				
	EDEV	IEDEV-1	IEDEV-2	IEDEV-3	IEDEV
F1	0.00E+00	0.00E+00	0.00E+00	0.00E+00	0.00E+00
	(0.00E+00)	(0.00E+00)	(0.00E+00)	(0.00E+00)	(0.00E+00)
F2	1.58E+04	1.35E+04	9.57E+03	1.34E+04	5.60E+03
	(1.01E+04)	(8.35E+03)	(7.38E+03)	(1.25E+04)	(6.91E+03)
F3	1.89E+05	1.92E+05	1.24E+05	1.76E+05	1.18E+05
	(7.23E+05)	(6.82E+05)	(3.58E+05)	(4.63E+05)	(2.76E+05)
F4	8.90E+00	8.16E+00	7.67E+00	1.17E+01	4.72E+00
	(7.16E+00)	(5.81E+00)	(9.34E+00)	(9.25E+00)	(7.28E+00)
F5	0.00E+00	0.00E+00	0.00E+00	0.00E+00	0.00E+00
	(0.00E+00)	(0.00E+00)	(0.00E+00)	(0.00E+00)	(0.00E+00)
F6	6.71E-01	6.82E-01	8.18E-01	5.84E-01	6.62E-01
	(1.35E+00)	(8.46E-01)	(9.81E-01)	(7.94E-01)	(1.31E+00)
F7	3.06E+00	2.81E+00	1.79E+00	2.91E+00	1.35E+00
	(5.16E+00)	(4.78E+00)	(2.31E+00)	(7.24E+00)	(6.34E+00)
F8	2.09E+01	2.10E+01	2.03E+01	2.07E+01	2.03E+01
	(6.00E-02)	(7.24E-02)	(5.27E-02)	(3.71E-03)	(5.41E-03)
F9	2.54E+01	2.48E+01	2.85E+01	2.84E+01	2.66E+01
	(2.27E+00)	(3.57E+00)	(3.39E+00)	(5.48E+00)	(2.13E+00)
F10	3.88E-02	3.25E-02	2.57E-02	2.67E-02	2.46E-02
	(2.42E-02)	(3.84E-02)	(1.86E-02)	(5.82E-03)	(4.27E-03)
F11	0.00E+00	0.00E+00	0.00E+00	0.00E+00	0.00E+00
	(0.00E+00)	(0.00E+00)	(0.00E+00)	(0.00E+00)	(0.00E+00)
F12	2.68E+01	2.73E+01	2.18E+01	2.59E+01	2.16E+01
	(7.17E+00)	(5.75E+00)	(8.64E+00)	(8.57E+00)	(6.73E+00)
F13	4.32E+01	4.39E+01	3.27E+01	3.97E+01	2.59E+01
	(1.71E+01)	(2.38E+01)	(1.37E+01)	(1.52E+01)	(1.37E+01)
F14	1.72E-01	1.64E-01	2.67E-01	1.68E-01	1.82E-01
	(1.07E-01)	(3.57E-01)	(1.58E-01)	(1.39E-01)	(2.25E-01)
F15	4.19E+03	4.28E+03	3.87E+03	3.53E+03	3.21E+03
	(4.96E+02)	(5.91E+02)	(5.61E+02)	(2.97E+02)	(1.78E+02)
F16	2.27E+00	1.76E+00	1.95E+00	1.81E+00	1.58E+00
	(3.79E-01)	(2.61E-01)	(4.27E-01)	(4.38E-01)	(3.64E-01)
F17	3.04E+01	3.04E+01	3.04E+01	3.04E+01	3.04E+01
	(2.54E-13)	(4.58E-12)	(1.57E-14)	(3.71E-13)	(8.49E-14)
F18	9.21E+01	9.35E+01	9.07E+01	7.24E+01	7.03E+01
	(8.61E+00)	(7.71E+00)	(5.67E+00)	(6.34E+00)	(5.75E+00)
F19	1.98E+00	1.75E+00	1.83E+00	1.52E+00	1.42E+00
	(1.56E-01)	(3.56E-01)	(2.43E-01)	(3.67E-01)	(5.73E-01)
F20	1.10E+01	1.08E+01	1.27E+01	1.08E+01	1.17E+01
	(3.99E-01)	(2.58E-01)	(4.97E-01)	(4.41E-02)	(3.96E-01)
F21	2.99E+02	2.86E+02	2.58E+02	2.75E+02	2.45E+02
	(7.01E+01)	(8.21E+01)	(6.61E+01)	(5.87E+01)	(8.63E+01)
F22	1.16E+02	1.28E+02	8.25E+01	1.37E+02	1.03E+02
	(2.72E+01)	(3.77E+01)	(3.78E+01)	(3.64E+01)	(2.95E+01)
F23	4.40E+03	4.46E+03	4.12E+03	4.37E+03	4.28E+03
	(7.69E+02)	(6.72E+02)	(3.97E+02)	(9.37E+02)	(6.52E+02)
F24	2.37E+02	2.46E+02	2.21E+02	2.16E+02	2.05E+02
	(3.22E+01)	(2.46E+01)	(1.58E+01)	(5.28E+01)	(1.83E+01)
F25	2.76E+02	2.65E+02	2.72E+02	2.75E+02	2.70E+02
	(6.85E+00)	(5.82E+02)	(4.23E+00)	(7.82E+00)	(5.85E+00)
F26	2.00E+02	2.00E+02	2.00E+02	2.00E+02	2.00E+02
	(8.13E-03)	(7.58E-03)	(5.48E-05)	(5.54E-05)	(8.32E-07)
F27	6.90E+02	5.68E+02	4.28E+02	6.22E+02	4.79E+02
	(2.19E+02)	(3.71E+02)	(3.41E+02)	(5.48E+02)	(6.62E+02)
F28	3.00E+02	3.00E+02	3.00E+02	3.00E+02	3.00E+02
	(0.00E+00)	(0.00E+00)	(0.00E+00)	(0.00E+00)	(0.00E+00)

**Table 3:** Friedman test ranks of EDEV and its variants proposed by us.

Algorithm	Ranking	Rank
IEDEV	1.9107	1
IEDEV-2	2.8036	2
IEDEV-3	2.9821	3
IEDEV-1	3.4464	4
EDEV	3.8571	5
Friedman p value	3.18E-06	

### Experiment for comparative comparison

Based on the CEC 2014 benchmark test suite and the CEC 2017 benchmark test suite, when dimensionality is set 30 and 100, our algorithm is compared with the nine peers listed above. Furthermore, we give convergence graphs. Details are listed below.

**Fig 1:**
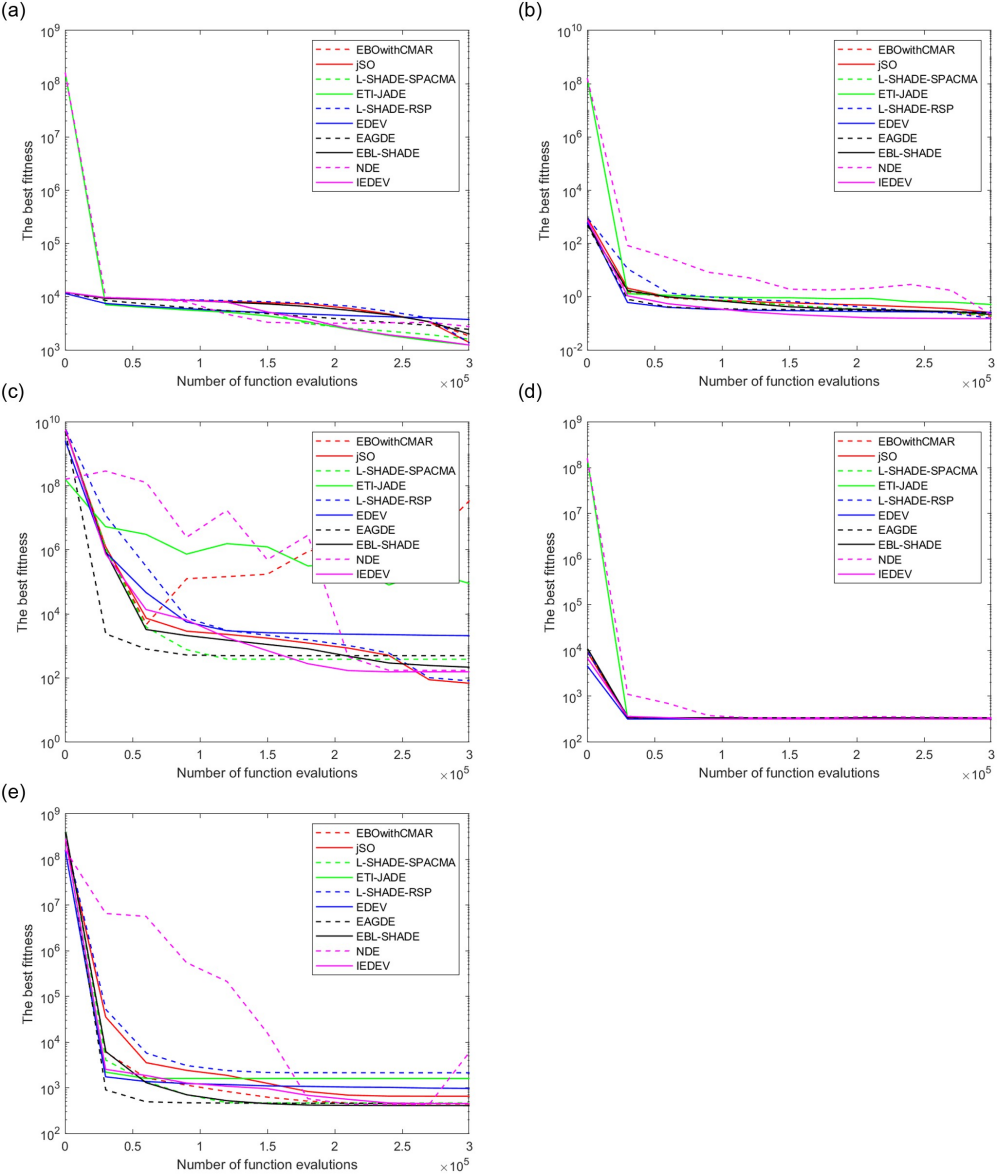
Convergence graphs of the ten algorithms for the five functions from the CEC 2014 suite. (a): F11, (b): F14, (c): F17, (d): F23, (e): F30.

The results of CEC 2014 functions are given in Tables 4 and 5. The Table 4 is for the comparison when dimensionality is set 30, while Table 5 is for 100. In Table 6, we lists Friedman test ranks based on the result in Tables 4 and 5. From the table, it can be seen that p-value is always less than 0.05. That is, there exists significant difference on performance. In Fig 1, convergence graph of the ten algorithms are plotted for five functions int the CEC 2014 sutie. For these functions, our algorithm never performs worse than the peers.

**Table 4:** Results for CEC 2014 suites with 30 in dimensionality.

Function	Average (standard deviation) of algorithms									
	EBOwith CMAR	jSO	L-SHADE-SPACMA	ETI-JADE	L-SHADE-RSP	EDEV	EAGDE	EBL-SHADE	NDE	IEDEV
F1	4.76E-06	0.00E+00	0.00E+00	7.09E+02	2.46E-14	3.26E+03	0.00E+00	0.00E+00	5.50E+02	8.70E+01
	(6.95E-06)+	(0.00E+00)+	(0.00E+00)+	(1.43E+03)−	(2.45E-14)+	(9.10E+03)−	(0.00E+00)+	(0.00e+00)+	(1.31E+03)−	(1.67E+02)
F2	2.18E-14	0.00E+00	0.00E+00	0.00E+00	0.00E+00	0.00E+00	0.00E+00	0.00E+00	0.00E+00	0.00E+00
	(1.22E-14)≈	(0.00E+00)≈	(0.00E+00)≈	(0.00E+00)≈	(0.00E+00)≈	(0.00E+00)≈	(0.00E+00)≈	(0.00e+00)≈	(0.00E+00)≈	(0.00E+00)
F3	1.33E-14	0.00E+00	0.00E+00	4.00E-04	0.00E+00	0.00E+00	0.00E+00	0.00E+00	0.00E+00	0.00E+00
	(2.45E-14)≈	(0.00E+00)≈	(0.00E+00)≈	(6.09E-04)−	(0.00E+00)≈	(0.00E+00)≈	(0.00E+00)≈	(0.00e+00)≈	(0.00E+00)≈	(0.00E+00)
F4	5.87E+01	0.00E+00	5.86E+01	2.11E+00	4.74E-14	6.86E-03	5.86E+01	5.86E+01	7.74E-04	8.37E-05
	(1.01E+00)−	(0.00E+00)+	(1.06E-14)−	(1.16E+01)−	(3.37E-14)+	(2.55E-02)−	(1.06E-14)−	(1.01e+00)−	(1.53E-03)≈	(7.37E-04)
F5	2.00E+01	2.08E+01	2.02E+01	2.00E+01	2.02E+01	2.04E+01	2.01E+01	2.01E+01	2.02E+01	2.01E+01
	(9.18E-06)+	(2.42E-01)−	(2.35E-02)≈	(1.91E-02)+	(4.93E-02)≈	(5.11E-02)−	(2.51E-02)≈	(3.42e-02)≈	(8.50E-02)≈	(5.30E-02)
F6	1.16E-01	8.52E-06	9.32E-02	4.90E-01	3.59E-02	5.21E-01	6.90E-01	0.00E+00	2.38E+00	5.63E-05
	(3.19E-01)−	(1.26E-05)+	(4.32E-01)−	(7.92E-01)−	(1.96E-01)−	(1.16E+00)−	(2.00E+00)−	(3.30e-01)+	(2.29E+00)−	(7.28E-04)
F7	0.00E+00	0.00E+00	0.00E+00	0.00E+00	0.00E+00	0.00E+00	0.00E+00	0.00E+00	0.00E+00	0.00E+00
	(0.00E+00)≈	(0.00E+00)≈	(0.00E+00)≈	(0.00E+00)≈	(0.00E+00)≈	(0.00E+00)≈	(0.00E+00)≈	(0.00e+00)≈	(0.00E+00)≈	(0.00E+00)
F8	9.47E-14	0.00E+00	0.00E+00	0.00E+00	4.11E-09	0.00E+00	0.00E+00	0.00E+00	2.32E+00	0.00E+00
	(4.31E-14)≈	(0.00E+00)≈	(0.00E+00)≈	(0.00E+00)≈	(7.73E-09)−	(0.00E+00)≈	(0.00E+00)≈	(5.24e-14)≈	(2.93E+00)−	(0.00E+00)
F9	1.38E+01	8.38E+00	5.39E+00	4.25E+01	9.13E+00	3.49E+01	7.82E+00	3.21E+00	5.12E+01	4.19E+00
	(2.93E+00)−	(1.87E+00)−	(2.13E+00)≈	(4.91E+00)≈	(1.98E+00)−	(5.46E+00)−	(1.55e+00)−	(1.40e+00)≈	(2.15E+01)−	(3.61E+00)
F10	2.01E-02	1.63E+00	1.11E-02	4.37E-02	6.32E+00	2.14E+00	2.78E-03	1.82E-12	4.37E+00	5.93E-02
	(2.08E-02)+	(1.17E+00)−	(1.52E-02)+	(3.43E-02)+	(2.37E+00)−	(9.49E+00)−	(7.20E-03)−	(1.48e-02)+	(2.27E+01)−	(7.56E-02)
F11	1.71E+03	1.31E+03	1.54E+03	1.15E+03	1.20E+03	2.51E+03	1.86E+03	1.39E+03	2.10E+03	8.50E+01
	(2.51E+02)−	(2.29E+02)−	(3.51E+02)−	(3.69E+02)−	(2.18E+02)−	(6.79E+02)−	(1.73E+02)−	(1.80e+02)−	(5.59E+02)−	(3.67E+02)
F12	9.29E-02	4.27E-01	2.25E-01	7.33E-02	2.39E-01	5.53E-01	2.42E-01	1.56E-01	1.55E-01	1.31E-01
	(3.65E-02)+	(3.53E-01)−	(3.27E-02)−	(2.77E-02)+	(5.01E-02)−	(1.66E-01)−	(3.89E-02)−	(4.07e-02)−	(1.02E-01)−	(6.18E-02)
F13	1.36E-01	1.35E-01	1.08E-01	1.39E-01	1.29E-01	1.94E-01	1.30E-01	8.29E-02	9.90E-02	9.51E-02
	(2.72E-02)−	(2.13E-02)−	(1.76E-02)+	(3.53E-02)+	(1.40E-02)≈	(3.36E-02)−	(1.82E-02)−	(1.16e-02)≈	(3.03E-02)≈	(4.62E-02)
F14	1.78E-01	2.24E-01	1.80E-01	1.63E-01	1.41E-01	1.75E-01	2.11E-01	1.52E-01	2.26E-01	1.52E-01
	(1.75E-02)−	(3.46E-02)−	(2.14E-02)−	(2.73E-02)−	(2.05E-02)+	(2.56E-02)−	(2.38E-02)−	(2.55e-02)≈	(3.66E-02)−	(2.85E-02)
F15	2.13E+00	2.22E+00	2.19E+00	2.61E+00	2.39E+00	4.03E+00	2.48E+00	1.99E+00	3.10E+00	2.03E+00
	(3.86E-01)−	(3.21E-01)−	(3.51E-01)≈	(5.72E-01)−	(3.62E-01)−	(4.78E-01)−	(3.43E-01)−	(2.47e-01)≈	(8.71E-01)−	(1.56E-01)
F16	9.80E+00	8.72E+00	8.77E+00	8.32E+00	8.31E+00	9.86E+00	9.92E+00	8.59E+00	1.00E+01	8.27E+00
	(4.71E-01)−	(7.48E-01)−	(6.34E-01)−	(5.62E-01)≈	(4.39E-01)≈	(4.19E-01)−	(4.75E-01)−	(4.63e-01)−	(7.26E-01)≈	(3.76E-01)
F17	2.72E+02	6.74E+01	3.81E+02	2.19E+04	6.67E+01	4.52E+03	3.67E+02	5.70E+01	1.91E+02	4.16E+03
	(1.12E+02)+	(1.52E+01)+	(1.45E+02)+	(6.44E+04)−	(3.88E+01)+	(8.35E+03)≈	(1.38E+02)+	(9.93e+01)+	(1.19E+02)+	(4.28E+03)
F18	1.29E+01	2.21E+00	1.36E+01	1.05E+02	2.70E+00	2.44E+01	9.70E+00	3.16E+00	8.58E+00	8.60E+00
	(7.35E+00)−	(1.18E+00)+	(5.23E+00)−	(2.09E+02)−	(1.54E+00)+	(1.58E+01)−	(4.25E+00)≈	(2.42e+00)+	(3.77E+00)≈	(3.67E+00)
F19	3.78E+00	1.99E+00	2.37E+00	3.83E+00	1.92E+00	3.65E+00	7.95E+00	3.43E+00	2.69E+00	2.16E+00
	(1.90E+00)−	(6.54E-01)+	(9.99E-01)−	(7.95E-01)−	(8.18E-01)+	(2.26E+00)−	(1.71E+00)−	(1.52e+00)−	(7.11E-01)−	(3.95E+00)
F20	3.65E+00	2.02E+00	3.80E+00	1.52E+02	1.94E+00	1.47E+01	4.13E+00	1.18E+00	5.30E+00	8.49E+00
	(1.73E+00)+	(6.92E-01)+	(1.86E+00)+	(1.09E+02)−	(6.42E-01)+	(3.60E+00)−	(1.45E+00)+	(1.22e+00)+	(1.70E+00)+	(2.57E+00)
F21	1.70E+02	2.62E+01	1.77E+02	2.06E+03	1.58E+01	3.62E+02	1.84E+02	2.17E+00	4.06E+01	1.58E+01
	(7.50E+01)−	(3.90E+01)−	(9.89E+01)−	(7.27E+03)−	(3.17E+01)≈	(1.71E+02)−	(9.88E+01)−	(8.50e+01)+	(5.14E+01)−	(6.82E+01)
F22	2.51E+01	2.91E+01	2.22E+01	1.03E+02	2.77E+01	1.03E+02	2.87E+01	2.42E+01	6.40E+01	9.23E+01
	(1.57E+00)+	(2.18E+01)+	(1.53E+00)+	(7.91E+01)≈	(2.21E+01)+	(6.14E+01)≈	(2.33E+00)+	(1.19e+00)+	(6.83E+01)+	(1.84E+01)
F23	3.36E+02	3.15E+02	3.36E+02	3.15E+02	3.15E+02	3.14E+02	3.36E+02	3.36E+02	3.15E+02	3.14E+02
	(8.44E-14)−	(1.09E-13)−	(0.00E+00)−	(1.11E-13)−	(5.78E-14)−	(1.57E-13)≈	(8.44E-14)−	(8.44e-14)−	(1.45E-13)−	(2.44E-14)
F24	1.97E+02	2.07E+02	2.01E+02	2.25E+02	2.06E+02	2.24E+02	2.01E+02	2.01E+02	2.21E+02	2.05E+02
	(1.84E+01)+	(1.02E+01)≈	(3.94E-02)≈	(2.42E+00)−	(1.02E+01)≈	(9.60E-01)−	(5.50E-02)+	(4.80e-02)+	(6.99E+00)−	(6.35E+00)
F25	2.00E+02	2.03E+02	2.01E+02	2.04E+02	2.03E+02	2.00E+02	2.02E+02	2.00E+02	2.03E+02	2.00E+02
	(9.77E-01))≈	(2.79E-02)≈	(1.24E+00)≈	(1.20E+00)−	(1.89E-02)−	(2.74E-02)≈	(1.41E+00)−	(1.05e+00)≈	(2.88E-01)−	(3.76E-02)
F26	1.00E+02	1.00E+02	1.00E+02	1.00E+02	1.00E+02	1.00E+02	1.00E+02	1.00E+02	1.00E+02	1.00E+02
	(2.51E-02))≈	(2.22E-02)≈	(2.33E-02)≈	(3.70E-02)≈	(1.56E-02)≈	(3.86E-02)≈	(1.41E-02)≈	(1.51e-02)≈	(5.82E-02)≈	(5.39E-02)
F27	3.01E+02	3.00E+02	3.02E+02	3.32E+02	3.01E+02	3.60E+02	3.02E+02	3.00E+02	4.00E+02	3.37E+02
	(1.74E+00)+	(1.71E-13)+	(2.83E+00)+	(4.50E+01)≈	(6.80E+00)+	(4.95E+01)−	(3.04E+00)+	(1.69e+00)+	(2.40E-01)−	(4.53E+01)
F28	4.14E+02	8.25E+02	4.19E+02	7.63E+02	8.18E+02	3.83E+02	4.21E+02	4.15E+02	8.26E+02	4.09E+02
	(2.16E+01)≈	(1.94E+01)−	(2.40E+00)+	(3.57E+01)−	(1.85E+01)−	(6.52E+00)+	(3.98E+00)−	(3.08e+00)−	(2.68E+01)−	(2.17E+01)
F29	4.33E+02	7.16E+02	4.96E+02	7.44E+02	7.15E+02	2.14E+02	4.32E+02	4.26E+02	6.46E+02	4.06E+02
	(1.37E+01)−	(2.48E+00)−	(1.16E+01)≈	(1.13E+02)−	(1.63E+00)−	(9.65E-01)+	(1.03E+01)−	(5.89e+00)−	(1.84E+02)−	(6.84E+00)
F30	4.43E+02	6.53E+02	4.63E+02	1.43E+03	2.01E+03	3.66E+02	4.43E+02	3.87E+02	5.62E+02	3.26E+02
	(1.33E+01)−	(2.19E+02)−	(5.51E+01)−	(4.60E+02)−	(7.02E+02)−	(1.07E+02)≈	(7.94E+01)−	(1.44e+01)−	(1.86E+02)−	(3.49E+02)
−	14	14	11	18	12	18	17	9	18	
+	9	9	8	4	9	2	6	10	3	
≈	7	7	11	8	9	10	7	11	9	

“+” or “−” denotes that the current result is significantly better or statistical worse than the result of our algorithm in terms of Wilcoxon’s rank sum test at a 0.05 significance level, respectively. Meanwhile, “≈” represents that there is no significant difference.

**Table 5:** Results for CEC 2014 suites with 100 in dimensionality.

Function	Average (standard deviation) of algorithms									
	EBOwith CMAR	jSO	L-SHADE-SPACMA	ETI-JADE	L-SHADE-RSP	EDEV	EAGDE	EBL-SHADE	NDE	IEDEV
F1	1.66E-02	1.37E+05	1.03E+04	1.39E+05	6.95E+04	1.07E+05	1.39E+05	6.68E+04	9.90E+05	1.68E+05
	(6.38e-03)+	(4.54E+04)≈	(8.52E+03)+	(1.07E+05)≈	(3.89E+04)+	(5.69E+04)+	(4.31E+04)≈	(5.49e+04)+	(2.69E+05))−	(8.57E+04)
F2	1.46E-04	0.00E+00	0.00E+00	1.56E-10	2.56E-13	2.39E-10	0.00E+00	1.42E-13	3.98E+03	0.00E+00
	(1.66e-04)−	(0.00E+00)≈	(0.00E+00)≈	(3.74E-10)≈	(1.04E-13)≈	(3.81E-10))−	(0.00E+00)≈	(8.50e-14)≈	(5.04E+03))−	(0.00E+00)
F3	3.16E-09	0.00E+00	0.00E+00	1.84E+02	4.26E-13	4.84E+00	0.00E+00	1.14E-13	1.26E+01	7.31E-11
	(3.76e-09)≈	(0.00E+00)+	(0.00E+00)≈	(1.56E+02))−	(1.37E-13)≈	(1.04E+01))−	(0.00E+00)≈	(1.97e-13)≈	(2.12E+01))−	(5.47E-11)
F4	1.77E+02	1.56E+02	2.05E+02	9.50E+01	1.77E+02	5.45E+01	1.85E+02	6.84E+01	1.62E+02	3.27E+01
	(6.04e+01)−	(2.80E+01))−	(1.04E+01)−	(4.44E+01))−	(3.28E+01))−	(6.85E+01))−	(2.95E+01)−	(3.84e+01)−	(2.95E+01))−	(3.98E+01)
F5	2.00E+01	2.08E+01	2.05E+01	2.00E+01	2.07E+01	2.08E+01	2.06E+01	2.05E+01	2.05E+01	2.00E+01
	(1.39e-06)≈	(3.15E-01))−	(3.67E-02)−	(6.98E-03)≈	(2.16E-01))−	(2.96E-01))−	(3.30E-02)−	(2.67e-02)−	(3.04E-01))−	(7.24E-04)
F6	1.14E+00	4.01E+00	7.75E-01	2.38E+01	1.31E+00	3.65E+01	1.23E+01	6.99E+00	6.18E+01	1.68E+00
	(1.10e+00)+	(1.76E+00))−	(6.24E-01)+	(9.22E+00))−	(1.17E+00)+	(4.21E+00))−	(2.62E+00)−	(2.71e+00)−	(1.38E+01))−	(1.49E+00)
F7	1.97E-13	0.00E+00	0.00E+00	9.04E-04	1.59E-13	1.40E-03	8.21E-04	1.14E-13	1.23E-03	7.56E-14
	(5.11e-14)≈	(0.00E+00)≈	(0.00E+00)≈	(2.86E-03))−	(5.66E-14)≈	(3.75E-03))−	(3.19E-03)−	(1.35e-03)≈	(4.13E-03))−	(2.57E-14)
F8	1.86E-01	4.20E-03	4.34E+00	1.14E-13	2.47E+00	0.00E+00	1.04E-01	6.04E-04	2.78E+01	0.00E+00
	(3.96e-01)−	(2.53E-03))−	(2.11E+00)−	(0.00E+00)≈	(1.35E+00))−	(0.00E+00)≈	(5.10E-02)−	(9.27e-04)−	(2.62E+01))−	(0.00E+00)
F9	5.16E+01	4.45E+01	1.16E+01	1.46E+02	3.70E+01	1.71E+02	5.72E+01	2.59E+01	8.95E+01	3.87E+01
	(1.25e+01)−	(6.75E+00))−	(2.75E+00)+	(2.47E+01))−	(8.51E+00)≈	(2.57E+01))−	(5.25E+00)−	(7.24e+00)+	(3.83E+01))−	(6.54E+00)
F10	8.56E+01	8.01E+01	1.72E+01	1.46E+02	2.64E+02	7.15E-01	6.68E+01	1.63E+01	3.83E+01	5.24E-01
	(1.50e+02)−	(2.59E+01))−	(3.45E+00)−	(2.68E+01))−	(5.27E+01))−	(2.27E-01))−	(1.16E+01)−	(4.69e+00)−	(1.51E+01))−	(7.27E-01)
F11	1.14E+04	1.01E+04	1.14E+04	9.41E+03	1.08E+04	1.11E+04	1.54E+04	1.20E+04	1.30E+04	1.06E+04
	(1.26e+03)≈	(7.02E+02)+	(1.12E+03)−	(8.88E+02)≈	(8.09E+02)≈	(3.50E+03)≈	(6.18E+02)−	(6.58e+02)−	(1.47E+03)≈	(9.14E+02)
F12	6.80E-02	4.60E-01	9.74E-01	1.04E-01	4.77E-01	8.30E-01	1.09E+00	7.61E-01	3.52E-01	3.48E-01
	(2.87e-02)−	(8.64E-02))−	(9.45E-02)−	(5.14E-02)+	(4.46E-02))−	(6.83E-01))−	(9.91E-02)−	(8.96e-02)−	(2.34E-01)≈	(5.57E-02)
F13	2.84E-01	3.09E-01	3.04E-01	3.22E-01	2.66E-01	3.64E-01	2.41E-01	1.87E-01	2.75E-01	3.37E-01
	(2.49e-02)≈	(4.29E-02)+	(2.45E-02)+	(5.38E-02)≈	(2.46E-02)+	(3.43E-02)≈	(1.82E-02)+	(1.67e-02)+	(4.64E-02)+	(2.47E-02)
F14	2.47E-01	2.10E+02	2.27E-01	1.14E-01	1.08E-01	2.09E-01	3.06E-01	2.65E-01	1.38E-01	1.52E-01
	(1.39e-02)−	(3.97E+01))−	(1.67E-02)−	(1.21E-02)+	(9.94E-03)+	(1.69E-02))−	(1.51E-02)−	(1.27e-02)−	(1.64E-02)+	(1.59E-02)
F15	1.06E+01	1.55E+01	1.27E+01	1.88E+01	1.68E+01	2.12E+01	1.92E+01	1.34E+01	1.18E+01	1.61E+01
	(3.02e+00)+	(1.93E+00)≈	(2.58E+00)+	(3.36E+00))−	(9.89E-01))−	(3.99E+00))−	(1.30E+00)−	(1.38e+00)+	(1.62E+00)+	(1.54E+00)
F16	4.06E+01	3.89E+01	3.88E+01	3.85E+01	3.89E+01	4.04E+01	4.05E+01	3.86E+01	4.23E+01	3.67E+01
	(1.33e+00)−	(7.05E-01))−	(6.52E-01)−	(1.84E+00))−	(4.19E-01))−	(5.51E-01))−	(5.36E-01)−	(6.83e-01)−	(1.76E+00)≈	(7.28E-01)
F17	3.88E+03	3.52E+03	3.30E+03	1.25E+04	3.99E+03	4.59E+04	5.21E+03	4.04E+03	9.81E+03	3.42E+04
	(6.92e+02)+	(7.34E+02)+	(4.47E+02)+	(4.79E+03)+	(5.40E+02)+	(1.89E+05))−	(7.45E+02)+	(7.04e+02)+	(4.94E+03)+	(2.51E+04)
F18	1.61E+02	2.17E+02	2.05E+02	6.40E+02	2.61E+02	3.05E+02	2.54E+02	2.08E+02	2.53E+02	2.11E+02
	(1.93e+01)+	(2.04E+01)≈	(2.61E+01)≈	(5.70E+02))−	(3.34E+01)≈	(6.52E+01))−	(2.78E+01)−	(2.03e+01)≈	(5.25E+01)−	(6.75E+01)
F19	1.04E+06	9.11E+01	5.93E+01	9.75E+01	9.12E+01	4.33E+01	7.04E+01	6.34E+01	9.23E+01	3.76E+01
	(7.94e+05)−	(1.19E+00))−	(1.37E+00)−	(1.76E+01))−	(1.24E+00))−	(2.87E+01)≈	(2.03E+00)−	(2.42e+00)−	(1.99E+00))−	(2.49E+01)
F20	3.38E+03	4.87E+01	3.07E+01	1.12E+03	3.52E+01	3.30E+02	1.73E+02	7.44E+01	3.15E+02	4.43E+02
	(4.84e+02)−	(1.14E+01)+	(7.22E+00)+	(1.21E+03))−	(8.30E+00)+	(9.56E+01)+	(4.49E+01)+	(2.73e+01)+	(6.82E+01)+	(1.28E+02)
F21	6.56E+02	9.04E+02	1.40E+03	4.22E+03	1.03E+03	7.47E+03	2.96E+03	1.08E+03	1.88E+03	5.47E+03
	(6.80e+01)+	(3.73E+02)+	(3.86E+02)+	(1.91E+03)+	(3.55E+02)+	(1.95E+04))−	(6.02E+02)+	(4.91e+02)+	(6.12E+02)+	(4.43E+04)
F22	1.89E+04	1.05E+03	8.66E+02	1.53E+03	1.10E+03	1.43E+03	7.37E+02	2.79E+02	1.46E+03	1.27E+03
	(5.45e+03)−	(2.43E+02)+	(3.96E+02)+	(3.84E+02)≈	(2.51E+02)+	(3.46E+02)≈	(2.07E+02)+	(1.73e+02)+	(5.28E+02))−	(5.41E+02)
F23	2.49E+04	3.48E+02	3.38E+02	3.48E+02	3.48E+02	3.45E+02	3.38E+02	3.38E+02	3.48E+02	3.06E+02
	(6.34e+03)−	(0.00E+00)−	(1.95E+00)−	(1.89E-13)−	(0.00E+00)−	(5.40E-12)−	(1.95E+00)−	(1.95e+00)−	(2.94E-12)+	(0.00E+00)
F24	2.00E+02	3.84E+02	2.03E+02	3.99E+02	3.87E+02	3.94E+02	2.03E+02	2.03E+02	3.80E+02	2.00E+02
	(2.05e+00)≈	(2.66E+00)−	(1.22E-01)−	(5.67E+00)−	(2.06E+00)−	(3.79E+00)−	(1.69E-01)−	(9.72e-02)−	(2.96E+00)−	(2.41E+00)
F25	1.39E+05	2.02E+02	2.14E+02	2.71E+02	2.16E+02	2.41E+02	2.17E+02	2.15E+02	2.29E+02	2.10E+02
	(3.47e+04)−	(4.79E+00)+	(2.08E-01)≈	(8.10E+00))−	(9.80E-01)−	(2.16E+01))−	(1.36E+00)≈	(1.27e+00)−	(1.26E+01)−	(3.57E+01)
F26	4.25E+04	2.00E+02	1.00E+02	2.00E+02	2.00E+02	2.00E+02	1.00E+02	1.00E+02	1.37E+02	2.00E+02
	(1.55e+03)−	(1.99E-13)≈	(2.25E-02)+	(1.69E-02)≈	(2.25E-02)≈	(1.59E-02)≈	(1.48E-02)+	(1.56e-02)+	(4.89E+01)+	(1.84E-02)
F27	2.09E+03	3.43E+02	3.15E+02	1.09E+03	3.08E+02	1.10E+03	5.04E+02	4.02E+02	6.77E+02	9.47E+02
	(2.59e+03)−	(3.14E+01)+	(1.41E+01)+	(1.15E+02)≈	(1.64E+01)+	(5.03E+02)≈	(5.99E+01)+	(4.95e+01)+	(8.92E+01)+	(3.57E+02)
F28	2.89E+04	2.14E+03	5.26E+02	2.30E+03	2.11E+03	6.80E+02	5.83E+02	5.31E+02	2.30E+03	6.68E+02
	(5.80e+04)−	(7.33E+01))−	(1.47E+01)+	(2.58E+02))−	(5.34E+01))−	(8.14E+01)≈	(1.60E+01)≈	(1.51e+01)+	(7.44E+01))−	(2.87E+01)
F29	4.40E+03	7.36E+02	9.27E+02	1.35E+03	7.74E+02	2.51E+02	9.31E+02	9.20E+02	1.22E+03	2.87E+02
	(6.91e+02)−	(3.06E+01))−	(6.72E+00)−	(2.03E+02))−	(5.94E+01))−	(5.09E+00)+	(5.99E+00)−	(7.08e+00)−	(2.49E+02))−	(4.26E+00)
F30	1.51E+11	4.73E+03	5.70E+03	8.36E+03	7.96E+03	2.67E+03	5.96E+03	3.83E+03	3.95E+03	2.33E+03
	(1.51e+10)−	(1.01E+03))−	(6.83E+02)−	(1.13E+03))−	(7.57E+02))−	(6.61E+02))−	(7.12E+02)−	(5.89e+02)−	(6.70E+02))−	(4.39E+02)
−	18	15	13	17	14	19	18	15	18	
+	6	9	12	4	9	3	7	11	9	
≈	6	6	5	9	7	8	5	4	3	

“+” or “−” denotes that the current result is significantly better or statistical worse than the result of our algorithm in terms of Wilcoxon’s rank sum test at a 0.05 significance level, respectively. Meanwhile, “≈” represents that there is no significant difference.

**Table 6:** Friedman test ranks for the CEC 2014 experiment.

Algorithm	30D	100D	Mean Ranking	Rank
EBL-SHADE	3.33	3.88	3.61	1
IEDEV	3.92	4.08	4.00	2
L-SHADE-SPACMA	5.23	3.85	4.54	3
jSO	5.23	4.85	5.04	4
L-SHADE-RSP	4.97	5.38	5.18	5
EAGDE	5.95	5.97	5.96	6
EBOwithCMAR	5.55	6.40	5.98	7
EDEV	6.80	6.70	6.75	8
NDE	7.00	6.85	6.93	9
ETI-JADE	7.02	7.03	7.03	10
Friedman p value	2.76E-08	2.85E-07		

The results of CEC 2017 functions are given in Tables 7 and 8. The former is for the comparison when dimensionality is set 30, while the latter is for 100. In Table 9, we lists Friedman test ranks based on the result in Tables 7 and 8. From the table, it can be seen that p-value is always less than 0.05. That is, there exists significant difference on performance.

**Table 7:** Results for CEC 2017 suites with 30 in dimensionality.

Function	Average (standard deviation) of algorithms									
	EBOwith CMAR	jSO	L-SHADE-SPACMA	ETI-JADE	L-SHADE-RSP	EDEV	EAGDE	EBL-SHADE	NDE	IEDEV
F1	0.00E+00	0.00E+00	0.00E+00	0.00E+00	0.00E+00	0.00E+00	0.00E+00	0.00E+00	0.00E+00	0.00E+00
	(0.00E+00)≈	(0.00E+00)≈	(0.00E+00)≈	(0.00E+00)≈	(0.00E+00)≈	(0.00E+00)≈	(0.00E+00)≈	(2.59e-15)≈	(0.00E+00)≈	(0.00E+00)
F2	0.00E+00	0.00E+00	0.00E+00	3.71E-13	1.42E-14	1.97E-12	0.00E+00	0.00E+00	4.84E-09	2.58E-14
	(0.00e+00)≈	(0.00E+00)≈	(0.00E+00)≈	(4.09E-13)≈	(1.94E-14)≈	(4.82E-12)≈	(0.00E+00)≈	(7.21e-15)≈	(2.46E-08)−	(3.57E-14)
F3	7.39E-14	0.00E+00	0.00E+00	6.59E+03	2.27E-14	5.96E-02	0.00E+00	0.00E+00	6.18E-04	0.00E+00
	(3.39e-14)≈	(0.00E+00)≈	(0.00E+00)≈	(6.52E+03)−	(2.83E-14)≈	(1.96E-01)−	(0.00E+00)≈	(2.79e-14)≈	(3.39E-03)−	(7.67E-14)
F4	5.93E+01	5.86E+01	5.86E+01	4.25E+01	5.86E+01	4.00E+00	5.86E+01	5.86E+01	5.91E+01	1.50E+00
	(1.92e+00)−	(2.08E-14)−	(0.00E+00)−	(2.43E+01)−	(2.11E-14)−	(1.72E+00)−	(1.49E-14)−	(3.17e-14)−	(1.70E+00)−	(8.96E-01)
F5	2.69E+00	8.79E+00	3.37E+00	2.21E+01	8.65E+00	3.27E+01	7.18E+00	3.11E+00	3.98E+01	5.46E+00
	(1.59e+00)+	(1.56E+00)−	(2.06E+00)+	(5.60E+00)−	(1.67E+00)−	(6.29E+00)−	(1.39E+00)≈	(1.48e+00)+	(1.98E+01)−	(2.37E+00)
F6	1.14E-13	1.14E-08	0.00E+00	1.14E-13	8.50E-08	1.14E-13	5.70E-09	1.14E-13	4.56E-09	1.14E-13
	(0.00e+00)≈	(3.46E-08)−	(0.00E+00)+	(0.00E+00)≈	(2.11E-07)−	(0.00E+00)≈	(2.55E-08)−	(2.50e-08)≈	(2.50E-08)−	(0.00E+00)
F7	3.35E+01	3.92E+01	3.40E+01	5.14E+01	4.02E+01	6.38E+01	3.86E+01	3.52E+01	5.96E+01	3.57E+01
	(8.12e-01)+	(1.70E+00)−	(9.41E-01)+	(4.62E+00)−	(1.65E+00)−	(5.60E+00)−	(1.13E+00)≈	(1.27e+00)≈	(1.03E+01)−	(3.27E+00)
F8	2.49E+00	9.33E+00	3.64E+00	2.30E+01	9.82E+00	3.33E+01	8.50E+00	5.03E+00	5.25E+01	7.63E+01
	(1.58e+00)−	(1.72E+00)+	(2.50E+00)+	(5.68E+00)+	(2.62E+00)+	(5.60E+00)+	(1.88E+00)+	(1.47e+00)+	(1.91E+01)+	(4.27E+00)
F9	0.00E+00	0.00E+00	0.00E+00	2.98E-03	0.00E+00	2.98E-03	0.00E+00	0.00E+00	0.00E+00	4.63E-05
	(0.00e+00)+	(0.00E+00)+	(0.00E+00)+	(1.63E-02)−	(0.00E+00)+	(1.63E-02)−	(0.00E+00)+	(0.00e+00)+	(0.00E+00)+	(5.73E-04)
F10	1.42E+03	1.51E+03	1.47E+03	1.27E+03	1.38E+03	2.81E+03	1.48E+03	9.54E+02	2.55E+03	7.28E+02
	(2.38e+02)−	(2.21E+02)−	(3.09E+02)−	(3.75E+02)−	(3.13E+02)−	(6.02E+02)−	(2.03E+02)−	(1.91e+02)−	(6.49E+02)−	(3.48E+02)
F11	1.11E+01	7.01E+00	1.07E+01	2.24E+01	1.27E+01	2.00E+01	2.83E+01	1.99E+00	1.35E+01	7.36E+00
	(2.01e+01)−	(1.48E+01)+	(1.75E+01)−	(2.39E+01)−	(2.17E+01)−	(1.70E+01)−	(2.89E+01)−	(2.75e+01)+	(1.44E+01)+	(3.76E+01)
F12	4.29E+02	1.92E+02	4.69E+02	1.35E+03	3.58E+02	1.24E+03	1.06E+03	3.41E+02	4.92E+02	5.39E+02
	(2.50e+02)≈	(1.03E+02)+	(2.45E+02)≈	(4.10E+02)−	(2.20E+02)+	(5.42E+02)−	(3.44E+02)−	(2.83e+02)≈	(2.28E+02)+	(1.43E+02)
F13	1.30E+01	1.51E+01	1.28E+01	2.77E+02	2.01E+01	9.06E+01	1.71E+01	9.95E-01	1.70E+01	1.57E+01
	(6.76e+00)+	(5.99E+00)≈	(6.51E+00)≈	(1.69E+01)−	(3.67E+00)−	(6.27E+01)−	(5.93E+00)≈	(5.62e+00)+	(8.07E+00)−	(8.48E+00)
F14	2.28E+01	2.23E+01	2.31E+01	1.33E+03	2.02E+01	3.09E+01	2.20E+01	5.63E-02	2.05E+01	2.08E+01
	(1.48e+00)−	(9.77E-01)−	(1.51E+00)−	(2.76E+03)−	(5.41E+00)+	(1.00E+01)−	(3.68E+00)≈	(4.35e+00)+	(1.00E+01)+	(1.00E+01)
F15	3.14E+00	1.03E+00	5.06E+00	5.56E+02	1.81E+00	1.53E+01	4.19E+00	3.00E-01	4.85E+00	2.42E-01
	(1.63e+00)−	(5.41E-01)−	(3.07E+00)−	(1.57E+03)−	(9.90E-01)−	(1.06E+01)−	(2.41E+00)−	(1.37e+00)−	(1.87E+00)−	(4.83E-01)
F16	6.88E+01	6.72E+01	6.13E+01	3.17E+02	2.44E+01	4.06E+02	9.61E+01	1.41E+01	2.66E+02	5.96E+01
	(7.60e+01)≈	(8.71E+01)≈	(8.12E+01)≈	(1.96E+02)−	(2.35E+01)+	(1.33E+02)−	(7.58E+01)−	(3.94e+01)+	(2.49E+02)−	(4.98E+01)
F17	3.20E+01	2.93E+01	3.19E+01	4.44E+01	3.38E+01	5.52E+01	3.58E+01	1.24E+01	6.04E+01	3.25E+01
	(6.65e+00)≈	(7.51E+00)+	(7.79E+00)≈	(4.24E+01)−	(7.78E+00)≈	(9.71E+00)−	(6.46E+00)−	(5.74e+00)+	(3.75E+01)−	(4.68E+00)
F18	2.25E+01	2.01E+01	2.37E+01	6.47E+03	2.08E+01	4.81E+01	2.34E+01	2.03E+01	2.31E+01	2.31E+01
	(1.34e+00)≈	(3.66E+00)+	(2.16E+00)≈	(1.98E+04)−	(5.60E-01)+	(2.92E+01)−	(4.39E+00)≈	(9.07e-01)≈	(5.64E+00)+	(5.90E+01)
F19	8.71E+00	4.41E+00	1.08E+01	6.56E+02	4.26E+00	1.49E+01	5.29E+00	2.90E+00	5.64E+00	2.14E+00
	(2.22e+00)−	(1.67E+00)−	(3.08E+00)−	(4.47E+03)−	(1.04E+00)−	(3.38E+00)−	(1.22E+00)−	(1.46e+00)−	(1.30E+00)−	(1.93E+00)
F20	3.61E+01	2.89E+01	8.12E+01	5.07E+01	2.80E+01	8.78E+01	3.40E+01	1.64E+01	7.59E+01	3.52E+01
	(6.63e+00)≈	(4.49E+00)+	(5.27E+01)−	(5.80E+01)≈	(6.12E+00)+	(6.07E+01)−	(5.81E+00-	(6.69e+00)+	(7.73E+01)−	(1.64E+01)
F21	2.03E+02	2.09E+02	2.07E+02	2.26E+02	2.10E+02	2.36E+02	2.08E+02	2.04E+02	2.39E+02	2.03E+02
	(1.50e+00)≈	(1.89E+00)−	(3.58E+00)−	(6.00E+00)−	(2.09E+00)−	(6.75E+00)−	(1.85E+00)−	(1.08e+00)−	(1.93E+01)−	(1.37E+00)
F22	1.00E+02	1.00E+02	1.00E+02	1.00E+02	1.00E+02	1.00E+02	1.00E+02	1.00E+02	1.00E+02	1.00E+02
	(0.00e+00)≈	(0.00E+00)≈	(2.12E-13)≈	(0.00E+00)≈	(0.00E+00)≈	(8.30E-14)≈	(0.00E+00)≈	(8.30e-14)≈	(0.00E+00)≈	(0.00E+00)
F23	3.53E+02	3.50E+02	3.56E+02	3.67E+02	3.55E+02	3.79E+02	3.52E+02	3.40E+02	3.77E+02	3.40E+02
	(4.52e+00)−	(3.52E+00)−	(3.27E+00)−	(7.03E+00)−	(4.15E+00)−	(1.30E+01)−	(3.33E+00)−	(3.07e+00)≈	(9.20E+00)−	(2.77E+00)
F24	4.25E+02	4.26E+02	4.29E+02	4.40E+02	4.29E+02	4.37E+02	4.27E+02	4.23E+02	4.51E+02	4.24E+02
	(2.31e+00)≈	(1.75E+00)≈	(3.04E+00)−	(5.60E+00)−	(2.78E+00)−	(8.71E+00)−	(2.25E+00)≈	(1.47e+00)≈	(1.20E+01)−	(3.57E+00)
F25	3.87E+02	3.87E+02	3.87E+02	3.87E+02	3.87E+02	3.78E+02	3.87E+02	3.87E+02	3.87E+02	3.78E+02
	(1.49e-02)−	(7.52E-03)−	(9.42E-03)−	(2.17E-01)−	(8.63E-03)−	(9.56E-02)≈	(2.03E-02)−	(2.56e-02)−	(5.74E-02)−	(6.44E-02)
F26	5.07E+02	9.29E+02	9.54E+02	1.13E+03	9.50E+02	9.17E+02	9.34E+02	8.20E+02	1.12E+03	8.02E+02
	(3.22e+02)+	(3.74E+01)−	(4.17E+01)−	(7.83E+01)−	(4.13E+01)−	(4.48E+02)−	(4.10E+01)−	(3.83e+01)−	(3.02E+02)−	(7.58E+01)
F27	5.04E+02	4.96E+02	5.06E+02	5.04E+02	5.02E+02	5.00E+02	5.00E+02	4.87E+02	4.94E+02	5.00E+02
	(4.17e+00)−	(6.58E+00)+	(5.13E+00)−	(8.00E+00)−	(6.07E+00)−	(1.54E-04)≈	(7.13E+00)≈	(6.75e+00)+	(9.56E+00)+	(7.61E-04)
F28	3.10E+02	3.00E+02	3.18E+02	3.47E+02	3.04E+02	3.48E+02	3.39E+02	3.00E+02	3.23E+02	3.01E+02
	(3.15e+01)≈	(2.56E-13)+	(4.16E+01)−	(5.51E+01)−	(2.08E+01)≈	(5.82E+01)−	(5.75E+01)−	(5.27e+01)≈	(4.74E+01)−	(2.37E+01)
F29	4.35E+02	4.34E+02	4.48E+02	4.30E+02	4.38E+02	4.06E+02	4.39E+02	4.17E+02	4.39E+02	4.12E+02
	(1.02e+01)−	(6.46E+00)−	(1.57E+01)−	(2.53E+01)−	(1.48E+01)−	(3.90E+01)+	(8.64E+00)−	(5.21e+00)−	(3.32E+01)−	(6.54E+01)
F30	2.00E+03	1.97E+03	2.00E+03	2.31E+03	1.97E+03	3.64E+02	1.99E+03	1.94E+03	2.01E+03	2.00E+02
	(4.67e+01)−	(2.40E+01)−	(7.32E+01)−	(1.53E+02)−	(3.77E+01)+	(6.63E+02)−	(5.25E+01)−	(6.42e+01)−	(5.90E+01)−	(5.74E+01)
−	12	14	16	24	16	22	17	9	21	
+	5	9	5	1	8	2	2	10	7	
≈	13	7	9	5	6	6	11	11	2	

“+” or “−” denotes that the current result is significantly better or statistical worse than the result of our algorithm in terms of Wilcoxon’s rank sum test at a 0.05 significance level, respectively. Meanwhile, “≈” represents that there is no significant difference.

**Table 8:** Results for CEC 2017 suites with 100 in dimensionality.

Function	Average (standard deviation) of algorithms									
	EBOwith CMAR	jSO	L-SHADE-SPACMA	ETI-JADE	L-SHADE-RSP	EDEV	EAGDE	EBL-SHADE	NDE	IEDEV
F1	1.22E+02	0.00E+00	0.00E+00	2.26E-10	9.55E-13	3.04E-10	0.00E+00	7.11E-14	1.68E+03	5.58E-11
	(1.32e+02)−	(0.00E+00)≈	(0.00E+00)≈	(5.92E-10)≈	(1.95E-12)≈	(4.15E-10)≈	(0.00E+00≈	(2.75e-11)≈	(2.28E+03)−	(7.83E-11)
F2	0.00E+00	1.42E+07	3.91E-07	4.72E+13	2.52E+03	1.77E+09	2.18E+01	1.42E-13	6.07E+39	5.49E+08
	(0.00e+00)+	(7.62E+07)≈	(2.13E-06)+	(2.58E+14)−	(8.08E+03)≈	(9.34E+09)−	(4.43E+01)+	(7.61e+05)+	(3.33E+40)−	(7.34E+08)
F3	3.22E-06	1.86E-06	0.00E+00	1.37E+05	3.92E-08	7.94E+01	4.86E-07	1.22E-07	1.87E+04	3.43E+01
	(5.87e-06)+	(1.23E-06)+	(0.00E+00)+	(1.01E+05)−	(2.68E-08)+	(1.70E+02)−	(5.23E-07)+	(1.69e-06)+	(8.30E+04)−	(1.50E+02)
F4	1.95E+02	1.91E+02	2.00E+02	8.59E+01	2.00E+02	2.77E+01	1.90E+02	7.48E+01	2.11E+02	2.39E+01
	(1.69e+01)−	(3.15E+01)−	(1.27E+01)−	(5.74E+01)−	(9.87E+00)−	(5.21E+01)≈	(2.50E+01)−	(3.87e+01)−	(2.47E+01)−	(4.68E+01)
F5	2.51E+03	4.35E+01	1.08E+01	1.42E+02	4.16E+01	1.82E+02	5.64E+01	3.26E+01	8.87E+01	4.28E+01
	(1.79e+02)−	(5.54E+00)≈	(2.60E+00)+	(2.58E+01)−	(7.94E+00)≈	(2.56E+01)−	(5.71E+00)≈	(4.58e+00)+	(1.80E+01)−	(2.73E+01)
F6	5.00E+01	3.68E-04	0.00E+00	2.05E-03	3.85E-05	3.28E-04	2.24E-02	1.49E-04	1.12E-05	1.56E-04
	(2.06e+01)−	(6.50E-04)−	(0.00E+00)+	(3.44E-03)−	(1.49E-04)+	(1.52E-03)−	(1.65E-02)−	(7.26e-03)≈	(1.02E-05)+	(8.49E-04)
F7	6.53E-12	1.46E+02	1.12E+02	2.66E+02	1.48E+02	3.01E+02	1.64E+02	1.31E+02	1.84E+02	1.27E+02
	(1.71e-12)+	(5.73E+00)−	(1.34E+00)+	(2.57E+01)−	(5.49E+00)−	(2.90E+01)−	(4.34E+00)−	(4.37e+00)−	(2.56E+01)−	(3.87E+00)
F8	8.79E+01	4.32E+01	1.09E+01	1.37E+02	4.20E+01	1.73E+02	5.64E+01	3.74E+01	9.51E+01	8.57E+01
	(1.53e+02)≈	(6.89E+00)+	(3.00E+00)+	(1.91E+01)−	(7.36E+00)+	(2.22E+01)−	(4.78E+00)+	(3.77e+00)+	(4.32E+01)≈	(1.35E+01)
F9	4.66E+03	8.45E-02	0.00E+00	5.47E+01	2.98E-03	9.46E+01	2.03E+00	1.14E-13	8.23E+00	6.59E+00
	(7.76e+02)−	(1.77E-01)+	(0.00E+00)+	(2.75E+01)−	(1.63E-02)+	(6.56E+01)−	(1.09E+00)+	(6.88e-01)+	(4.54E+00)≈	(3.67E+00)
F10	3.51E+04	9.85E+03	9.89E+03	8.91E+03	1.02E+04	1.09E+04	1.15E+04	8.96E+03	1.15E+04	9.82E+03
	(1.34e+03)−	(5.81E+02)≈	(8.19E+02)≈	(1.31E+03)≈	(8.43E+02)−	(2.15E+03)≈	(5.59E+02)−	(5.46e+02)≈	(1.46E+03)≈	(4.34E+02)
F11	9.97E+02	1.08E+02	4.91E+01	5.43E+02	7.86E+01	6.38E+02	5.93E+02	1.41E+02	5.01E+02	5.26E+02
	(3.22e+02)−	(3.03E+01)+	(2.49E+01)+	(2.38E+02)−	(2.80E+01)+	(2.77E+02)≈	(9.87E+01)≈	(1.30e+02)+	(1.60E+02)≈	(6.76E+02)
F12	3.13E+11	1.66E+04	4.63E+03	5.48E+03	1.32E+04	1.58E+04	2.30E+04	8.76E+03	1.69E+05	1.35E+04
	(4.16e+10)−	(6.12E+03)−	(6.80E+02)+	(1.66E+03)+	(6.18E+03)≈	(6.44E+03)−	(1.42E+04)−	(7.97e+03)+	(9.22E+04)−	(4.94E+03)
F13	4.84E+09	1.44E+02	1.28E+02	2.10E+03	1.69E+02	1.11E+03	4.15E+02	1.13E+02	3.57E+02	8.61E+02
	(9.28e+08)−	(3.36E+01)+	(2.97E+01)+	(1.08E+03)−	(3.69E+01)+	(9.57E+02)−	(1.97E+02)+	(7.47e+01)+	(1.25E+02)+	(4.82E+01)
F14	1.55E+08	6.39E+01	7.04E+01	6.22E+02	5.92E+01	1.23E+04	2.93E+02	1.81E+02	1.48E+02	5.76E+01
	(9.82e+07)−	(1.21E+01)≈	(9.15E+00)−	(1.41E+02)−	(9.86E+00)−	(4.81E+04)−	(5.48E+01)−	(2.89e+01)−	(3.42E+01)−	(2.81E+01)
F15	3.68E+09	1.51E+02	1.02E+02	4.69E+02	1.80E+02	3.17E+02	2.76E+02	1.60E+02	2.85E+02	1.46E+02
	(1.27e+09)−	(2.68E+01)−	(2.27E+01)+	(1.38E+02)−	(3.99E+01)−	(1.24E+02)−	(5.66E+01)−	(5.07e+01)−	(5.98E+01)−	(2.34E+01)
F16	2.59E+04	1.90E+03	1.29E+03	2.05E+03	1.55E+03	2.35E+03	1.80E+03	8.40E+02	2.19E+03	1.92E+03
	(4.76e+03)−	(2.80E+02)≈	(4.61E+02)+	(4.00E+02)≈	(3.25E+02)+	(3.71E+02)−	(2.75E+02)≈	(2.11e+02)+	(4.34E+02)−	(6.12E+02)
F17	3.29E+03	1.31E+03	9.25E+02	1.03E+03	1.06E+03	1.63E+03	1.31E+03	6.38E+02	1.60E+03	1.07E+03
	(5.77e+02)−	(1.76E+02)−	(3.39E+02)≈	(2.55E+02)≈	(3.03E+02)≈	(2.29E+02)−	(1.84E+02)−	(1.90e+02)+	(4.93E+02)−	(3.18E+02)
F18	1.67E+02	1.83E+02	1.35E+02	2.18E+03	2.02E+02	2.23E+03	2.72E+02	1.26E+02	3.64E+02	1.98E+02
	(3.42e+01)≈	(2.34E+01)≈	(3.38E+01)+	(1.60E+03)−	(4.44E+01)≈	(1.65E+03)−	(4.88E+01)≈	(4.53e+01)+	(9.59E+01)−	(4.18E+03)
F19	8.36E+05	1.03E+02	7.47E+01	1.55E+03	1.20E+02	4.27E+02	1.95E+02	1.19E+02	1.75E+02	1.12E+02
	(6.77e+05)−	(2.33E+01)≈	(1.31E+01)≈	(1.23E+03)−	(2.57E+01)−	(4.05E+02)−	(3.24E+01)−	(2.14e+01)≈	(4.43E+01)≈	(3.65E+02)
F20	3.42E+03	1.34E+03	1.39E+03	1.07E+03	1.29E+03	1.51E+03	1.77E+03	1.21E+03	2.08E+03	1.16E+03
	(3.94e+02)−	(2.43E+02)−	(3.36E+02)−	(5.27E+02)+	(3.45E+02)−	(2.91E+02)−	(1.89E+02)−	(1.84e+02)≈	(3.29E+02)−	(2.57E+02)
F21	6.60E+02	2.65E+02	2.41E+02	3.76E+02	2.68E+02	3.96E+02	2.76E+02	2.38E+02	3.12E+02	2.58E+02
	(5.39e+01)−	(6.81E+00)−	(4.12E+00)+	(6.17E+01)−	(8.89E+00)−	(2.59E+01)−	(8.91E+00)−	(7.28e+00)+	(2.20E+01)−	(8.54E+00)
F22	1.73E+04	1.06E+04	9.89E+03	1.33E+04	1.08E+04	1.32E+04	1.24E+04	1.02E+04	1.32E+04	1.02E+04
	(1.04e+03)−	(7.45E+02)−	(1.20E+03)≈	(5.16E+03)−	(7.53E+02)−	(4.93E+03)−	(5.45E+02)−	(4.96e+02)≈	(8.34E+02)≈	(6.86E+02)
F23	2.62E+04	5.71E+02	5.82E+02	1.24E+03	5.62E+02	6.84E+02	5.74E+02	5.56E+02	6.46E+02	5.42E+02
	(3.79e+03)−	(1.03E+01)−	(7.17E+00)−	(4.72E+01)−	(1.00E+01)−	(3.52E+01)−	(1.21E+01)−	(9.28e+00)−	(2.46E+01)−	(2.61E+01)
F24	2.00E+02	9.00E+02	9.14E+02	2.14E+02	9.07E+02	1.10E+03	9.17E+02	8.90E+02	9.71E+02	9.00E+02
	(2.68e+00)+	(8.13E+00)≈	(2.11E+01)−	(3.45E+01)+	(9.49E+00)−	(4.70E+01)−	(9.77E+00)−	(9.22e+00)+	(2.22E+01)−	(7.65E+00)
F25	1.54E+05	7.31E+02	6.93E+02	8.14E+02	7.01E+02	7.70E+02	7.47E+02	6.98E+02	7.38E+02	7.09E+02
	(5.54e+04)−	(3.63E+01)−	(4.74E+01)≈	(5.62E+01)≈	(4.27E+01)≈	(6.08E+01)−	(3.39E+01)−	(3.00e+01)≈	(4.58E+01)−	(4.66E+01)
F26	4.30E+04	3.25E+03	3.13E+03	7.53E+03	3.24E+03	4.91E+03	3.39E+03	3.05E+03	3.83E+03	4.27E+03
	(1.75e+03)−	(9.40E+01)+	(9.23E+01)+	(4.49E+03)−	(1.00E+02)+	(3.16E+02)−	(9.02E+01)+	(7.02e+01)+	(1.97E+02)+	(2.21E+02)
F27	2.98E+03	5.87E+02	5.99E+02	1.79E+03	6.02E+02	6.93E+02	6.45E+02	5.86E+02	6.53E+02	6.03E+02
	(2.96e+03)−	(2.28E+01)+	(2.07E+01)≈	(1.81E+02)−	(1.46E+01)≈	(1.76E+02)−	(2.47E+01)−	(1.80e+01)+	(2.87E+01)−	(3.64E+01)
F28	1.04E+04	5.26E+02	5.12E+02	5.00E+02	5.25E+02	4.19E+02	5.24E+02	5.03E+02	5.68E+02	4.36E+02
	(3.78e+04)−	(2.50E+01)−	(1.50E+01)−	(2.06E+01)−	(2.64E+01)−	(8.65E+01)+	(2.03E+01)−	(2.61e+01)−	(3.34E+01)−	(3.28E+01)
F29	4.29E+03	1.27E+03	1.52E+03	1.49E+03	1.44E+03	1.76E+03	1.35E+03	8.61E+02	1.67E+03	1.64E+03
	(6.03e+02)−	(1.71E+02)+	(3.60E+02)≈	(2.90E+02)+	(1.79E+02)≈	(3.27E+02)−	(1.82E+02)+	(1.55e+02)+	(3.54E+02)≈	(4.66E+02)
F30	1.57E+11	2.31E+03	2.39E+03	6.23E+03	2.55E+03	8.81E+02	2.45E+03	2.16E+03	2.51E+03	7.24E+02
	(2.06e+10)−	(1.22E+02)−	(2.06E+02)−	(5.82E+03)−	(2.27E+02)−	(2.60E+02)≈	(1.74E+02)−	(1.32e+02)−	(1.45E+02)−	(3.57E+02)
−	24	13	7	21	13	24	18	7	20	
+	4	8	15	4	8	1	7	16	3	
≈	2	9	8	5	9	5	5	7	7	

“+” or “−” denotes that the current result is significantly better or statistical worse than the result of our algorithm in terms of Wilcoxon’s rank sum test at a 0.05 significance level, respectively. Meanwhile, “∼” represents that there is no significant difference.

**Table 9:** Friedman test ranks for the CEC 2017 experiment.

Algorithm	30D	100D	Mean Ranking	Rank
EBL-SHADE	2.62	2.55	2.58	1
IEDEV	3.73	4.23	3.98	2
L-SHADE-SPACMA	5.52	2.85	4.18	3
jSO	4.38	4.37	4.38	4
L-SHADE-RSP	5.35	4.38	4.87	5
EAGDE	5.87	6.03	5.95	6
EBOwithCMAR	4.73	8.50	6.62	7
NDE	7.45	7.43	7.44	8
ETI-JADE	7.92	6.97	7.44	9
EDEV	7.43	7.68	7.56	10
Friedman p value	0.00E+00	0.00E+00		

According to Table 4, in term of Wilcoxon’s rank sun test, our algorithm performs worse than EBL-SHADE for the CEC 2014 suite with 30 in dimensionality but better than all the other peers. According to Table 5, in term of Wilcoxon’s rank sun test, our algorithm defeats all the peers for the CEC 2014 suite with 100 in dimensionality. It can be seen from Table 6 that, in term of Friedman test, our algorithm ranks second. That is, our algorithm defeats all the peers except EBL-SHADE. Fig 1 demonstrates that our algorithm does not performs outstanding at all at the initial stage but show make a greater progress than most of the peers in the latter stage.

According to Table 7, in term of Wilcoxon’s rank sun test, our algorithm performs worse than EBL-SHADE for the CEC 2017 suite with 30 in dimensionality but better than all the other peers. According to Table 8, in term of Wilcoxon’s rank sun test, our algorithm performs worse than L-SHADE-SPACMA and EBL-SHADE for the CEC 2017 suite with 100 in dimensionality but better than all the other peers. It can be seen from Table 9 that, in term of Friedman test, our algorithm ranks second. That is, our algorithm defeats all the peers except EBL-SHADE.

### Discussion

In fact, the peers selected by us are good performers in the CEC competitions on real parameter single objective optimization among population-based metaheuristics, state-of-the-art algorithms, or up-to-date algorithms. According to the results of our experiments, our IEDEV is competitive among DE algorithms. Based on EDEV, we integrate three measures. Firstly, individuals in optional external archive of the constituent algorithm JADE is collected from a wider range to maintain diversity. Then, the fiercer competition among the three constituent DE algorithms than ever brought by the ETI control contributes to improvement on solution. Moreover, LPSR further improves overall performance. Hence, our algorithm performs much better than EDEV and becomes so competitive.

## Conclusion

Most existing measures for improving DE algorithm need be revised for fitting ensemble DE algorithm. In this paper, we propose IEDEV—an ensemble DE algorithm with three measures. To obtain the algorithm, firstly, we extend the collecting range of optional external archive of JADE—one of the constituent algorithm in EDEV. Then, we revise the ETI control and implement it into algorithm. Finally, LPSR is used by us. In our experiments, we compare our IEDEV with nine peers. Experimental results show that our algorithm is competitive.

Our IEDEV shows that, measures for DE algorithm may be used in ensemble DE algorithm although revision is needed in many occasions. We will continue our research in the future to consider more techniques.
